# A Case of Wallenberg Syndrome Presenting With Thunderclap Headache and Delayed Diagnosis Due to Negative Findings on Two MRI Examinations

**DOI:** 10.7759/cureus.82236

**Published:** 2025-04-14

**Authors:** Yoshiro Nozaki, Mitsuharu Yamamoto, Koichiro Demura, Yuto Sakakibara

**Affiliations:** 1 Neurosurgery, Toyokawa City Hospital, Toyokawa, JPN

**Keywords:** 3-tesla mri, case reports, medulla oblongata infarction, thunderclap headache, wallenberg’s syndrome

## Abstract

Wallenberg syndrome is a lateral medullary infarction characterized by various neurological symptoms, including sensory disturbances, vestibular symptoms, and autonomic dysfunction. However, its initial presentation can vary, and the possibility of false-negative magnetic resonance imaging (MRI) findings can make diagnosis challenging. We report the case of a 65-year-old woman who developed a sudden, severe frontal headache and presented to the emergency department 2 hours later. She complained of headache accompanied by nausea; however, no other neurological abnormalities were observed. An initial brain MRI and a follow-up MRI performed 8 hours later showed no abnormalities; however, her headache persisted, and she developed vertigo, right facial sensory disturbance, and dysphagia, prompting reevaluation. MRI revealed an acute infarction in the right lateral medulla, leading to a diagnosis of Wallenberg syndrome. Throughout the course, no findings suggestive of vertebral artery dissection were observed. The patient was treated with dual antiplatelet therapy and continued rehabilitation. Due to persistent dysphagia, she was transferred to a rehabilitation hospital on the 27th hospital day. This rare case suggests that thunderclap headaches can be an initial manifestation of Wallenberg syndrome. Furthermore, our case highlights that even if the initial MRI is negative, Wallenberg syndrome should be considered in the differential diagnosis, particularly in patients with progressive neurological symptoms.

## Introduction

Wallenberg syndrome, a subtype of cerebral infarction, occurs due to infarction in the dorsolateral medulla caused by occlusion of the posterior inferior cerebellar artery (PICA), a branch of the vertebral artery [1]. Its diverse mechanisms include atherosclerosis, vertebral artery dissection, and embolism [2]. Wallenberg syndrome is known to present with a variety of symptoms, including sensory disturbances in the face, trunk, and limbs; vestibular and cerebellar symptoms, such as dizziness, imbalance, and ataxia; autonomic dysfunction represented by Horner’s syndrome; and bulbar symptoms, such as dysphagia, hoarseness, and hiccups. However, the combination of these symptoms and their timing of onset vary among cases [3,4]. In addition, early-stage magnetic resonance imaging (MRI) may fail to detect the lesion, often leading to a delayed diagnosis [5-7].

We report a case of Wallenberg syndrome presenting solely with thunderclap headache as the initial symptom, in which two MRI scans failed to provide a diagnosis and no clear signs of arterial dissection.

## Case presentation

The patient was a 65-year-old woman with a history of cerebral infarction accompanied by mild left-sided incomplete hemiparesis and was receiving aspirin 100 mg at a nearby medical institution. She had no history of primary headache disorders or any recent episodes of trauma. One night, she developed a sudden and extremely severe frontal headache and was transported by ambulance to the hospital approximately 2 hours later. On arrival, the Glasgow Coma Scale (GCS) score was 15 (E4V5M6), and she could walk independently. She had no dizziness or imbalance, and both nuchal rigidity and jolt accentuation were negative. No abnormalities in eye movements, diplopia, or nystagmus were observed. In addition, Barre’s sign was negative in both the upper and lower limbs, and there was no worsening of the pre-existing mild left-sided hemiparesis.

**Figure 1:**
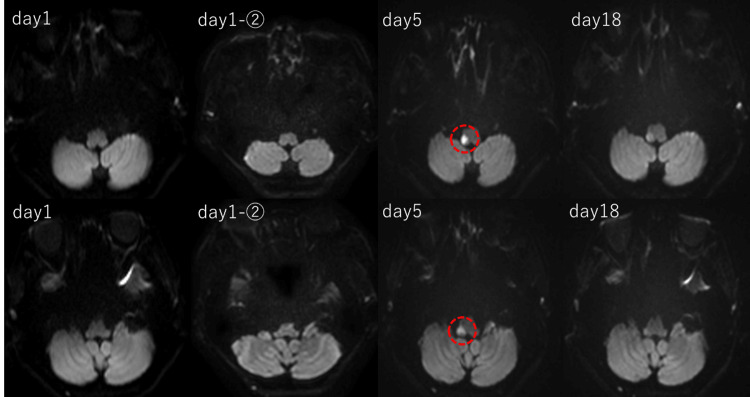
The temporal changes are shown with diffusion-weighted imaging (DWI) (B-value = 1000 s/mm²). The infarct in the right lateral medulla was not visualized on the initial two magnetic resonance imaging (MRI) scans and was first identified on MRI performed on day 5 after onset. MRI findings suggest that the branches of the right posterior inferior cerebellar artery (PICA) supplying the cerebellar hemisphere were preserved, while those supplying the medulla were involved.

**Figure 2:**
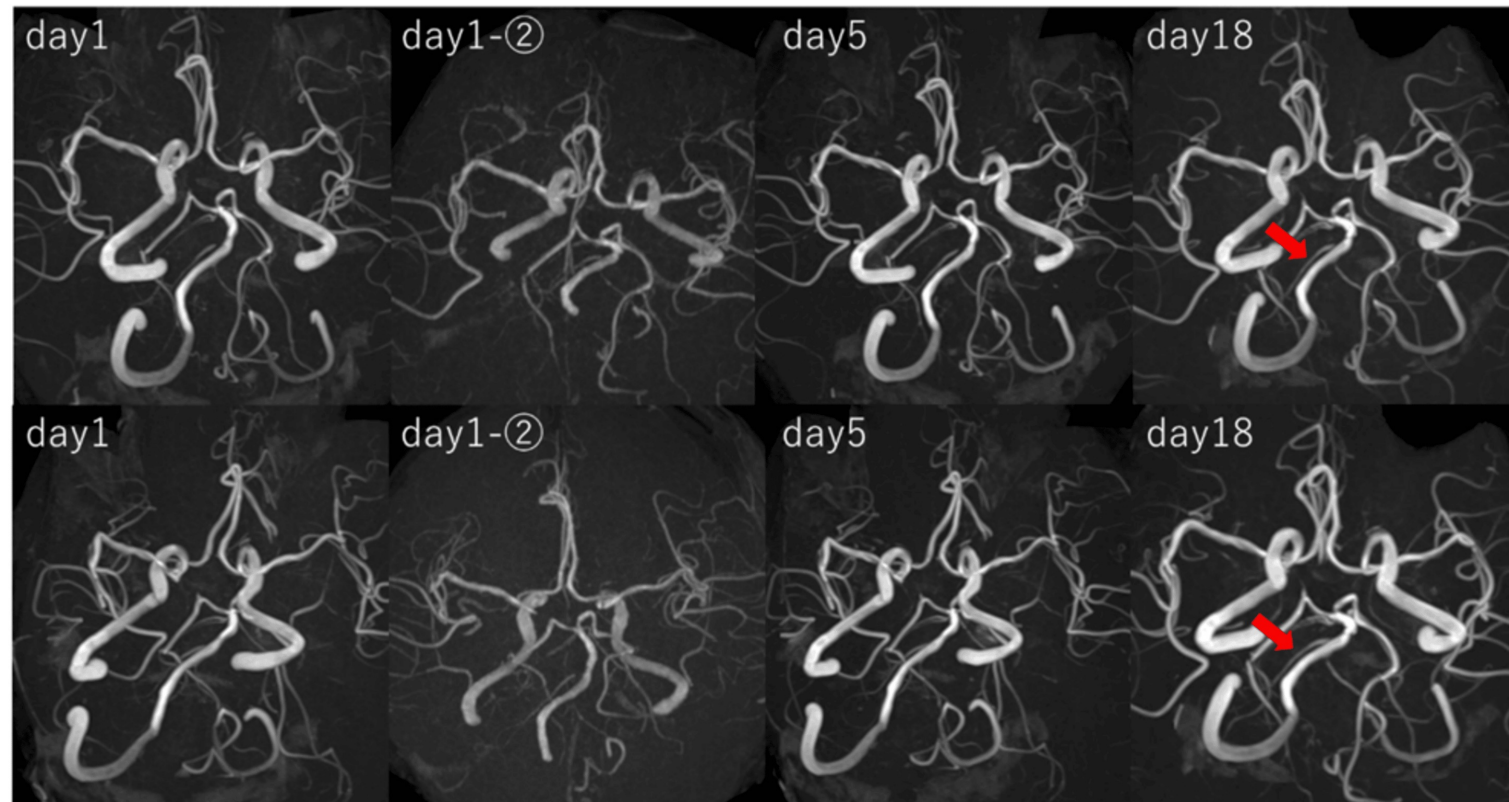
Magnetic resonance angiography (MRA) images taken at different time points. No findings suggestive of dissection were observed in the right vertebral artery, and no temporal morphological changes were noted. The right posterior inferior cerebellar artery (PICA) was consistently not visualized (arrow indicates the developed anterior inferior cerebellar artery (AICA)).

The headache was persistent; by the time of presentation, it had become a generalized headache affecting the entire head. There were no apparent prodromal symptoms or episodes of photophobia, osmophobia, and phonophobia. She reported nausea without vomiting; however, no other obvious neurological abnormalities were observed. Head computed tomography (CT) and 3-tesla (3T) MRI (Ingenia, Philips Medical Systems, Best, The Netherlands) revealed no apparent abnormalities, and there were no findings suggestive of dissection from the bilateral vertebral arteries to the basilar artery (Figures 1, 2). The right PICA was not visualized on magnetic resonance angiography (MRA; Figure 2).

The patient was admitted to the hospital and underwent a second 3T MRI (Magnetom Skyra, Siemens Healthineers AG, Munich, Germany) the following day, 8 hours after the initial MRI. However, similar to the previous examination, no findings suggestive of cerebral infarction or arterial dissection were observed (Figure 2). Lumbar puncture and catheter angiography were considered to rule out subarachnoid hemorrhage; however, these procedures were not performed due to concerns about invasiveness and potential complications. Immediately afterward, the patient experienced severe vertigo with persistent headache and nausea. Eye movements were normal, with no signs of diplopia or nystagmus. Bilateral perioral sensory impairment was observed, but there was no worsening of the pre-existing mild hemiparesis in the left upper and lower limbs. Furthermore, no obvious dysarthria or dysphagia was noted.

The characteristics of the headache remained unchanged, and head CT revealed no significant abnormalities. Initially, there were few findings suggestive of central vertigo, and peripheral vertigo disorders, including benign paroxysmal positional vertigo (BPPV), were suspected; however, from the second day of illness, the patient began to complain of hypoesthesia in the right side of the face and dysphagia. On the fifth day of illness, an MRI (3T Skyra, Siemens) revealed a cerebral infarction in the right lateral medulla (Figure 1). MRA showed no changes in vascular morphology compared to the initial imaging (Figure 2). At the time of diagnosis, the symptoms included headache, nausea, vertigo, sensory impairment in the right side of the face, dysphagia, and mild hoarseness, which were consistent with Wallenberg syndrome. As the patient was already taking aspirin 100 mg, clopidogrel 75 mg was added to the treatment.

Electrocardiography, transthoracic echocardiography, and blood tests were performed to investigate the cause of the cerebral infarction. Electrocardiography showed sinus rhythm, and echocardiography revealed no evidence of significant valvular heart disease, intracardiac thrombus, or cardiac tumors. Blood tests, including coagulation factors, showed no abnormalities. There were no clinical findings suggestive of connective tissue diseases, such as tall stature or skin hyperextensibility, nor were there any signs of vasculitis, such as fever, weight loss, or renal dysfunction. On the 18th day of illness, a follow-up MRI (3T Skyra, Siemens) was performed, confirming that the infarct lesion had not expanded. Furthermore, there were no changes in vascular morphology or visualization on MRA (Figures 1, 2). Rehabilitation was continued, and while the headache improved, vertigo and dysphagia persisted. On the 27th day of illness, the patient was transferred to a rehabilitation hospital.

## Discussion

This case of Wallenberg syndrome demonstrated a rare clinical course that initially presented with a thunderclap headache with no apparent lesion on the initial MRI. In addition, MRA did not reveal any apparent signs of dissection, including the pearl and string sign. We considered the possibility that the thunderclap headache occurred in a non-dissecting manner. In general, the differential diagnosis of thunderclap headache includes subarachnoid hemorrhage, including sentinel bleeding, in addition to carotid or vertebral artery dissection, reversible cerebral vasoconstriction syndrome (RCVS), pituitary apoplexy, and meningitis [8]. The typical early symptoms of Wallenberg syndrome include headache, vertigo, balance disturbances, dysphagia, and hoarseness; however, thunderclap headache as the sole initial symptom is rare [1]. Vertebral artery dissection, a known cause of Wallenberg syndrome, can present with severe headache as an initial symptom [9]. In this case, repeated MRA consistently showed no findings suggestive of vertebral artery dissection or temporal changes in vascular morphology (Figure 2). Additionally, the right PICA was not visualized in the initial examination. Dissection of the main trunk of the PICA was considered a possible cause of Wallenberg syndrome; however, subsequent MRI confirmed that the infarction was localized to the right lateral medulla. If the right PICA dissection had caused the vessel to become non-visualized, the infarct would likely have been detected earlier and extended to the inferior surface of the cerebellum. Therefore, this possibility was considered unlikely. In this case, the right anterior inferior cerebellar artery (AICA) was well-developed, suggesting that the PICA was hypoplastic (Figure 2). Additionally, other potential causes of thunderclap headache could be ruled out based on clinical findings and MRI results. In this case, repeated MRI scans showed no evidence of subarachnoid hemorrhage or an unruptured cerebral aneurysm. In addition, the other listed differential diagnoses were considered unlikely as their typical clinical courses were inconsistent with this case and were further ruled out by repeated MRI findings.

Several mechanisms could potentially explain the association between thunderclap headache and Wallenberg syndrome in the absence of arterial dissection. One possible mechanism is that lateral medullary infarction affected the trigeminal pain pathways, leading to a sudden and severe headache. Lambru et al. reported a case of Wallenberg syndrome complicated by trigeminal neuralgia (TN) and short-lasting unilateral neuralgiform headache attacks with conjunctival injection and tearing (SUNCT) [10]. TN is triggered by abnormalities in the wide dynamic range neurons of the spinal trigeminal nucleus, particularly in its caudal subnucleus. SUNCT, on the other hand, arises from the hyperexcitability of the trigemino-hypothalamic tract, in which the spinal trigeminal nucleus plays a role. In both conditions, damage to the spinal trigeminal nucleus serves as the underlying trigger. Warren et al. reported a case in which a lateral medullary infarction caused ipsilateral TN and glossopharyngeal neuralgia. They identified infarct lesions in the spinal trigeminal nucleus and tract, as well as the solitary nucleus, and proposed that ischemic lesions in the lateral medulla could affect the spinal trigeminal tract and nucleus, contributing to pain [11]. Additionally, since the medulla contains autonomic regulatory centers, infarction-induced autonomic dysfunction may have altered the vascular tone, potentially triggering the thunderclap headache. Pakeerathan et al. reported a case of Wallenberg syndrome presenting with thunderclap headache and transient high-grade atrioventricular block [12]. The patient experienced a thunderclap headache accompanied by bulbar palsy symptoms and vertigo. An infarct lesion was identified in the solitary nucleus of the medulla; however, there was no mention of the presence or absence of vascular dissection. Furthermore, although rare, Zhu et al. reported a case of symptomatic migraine associated with visual disturbances related to a brainstem lesion [13]. Disruption of ascending neural activity from the spinal trigeminal nucleus on the lesion side and the spinal dorsal horn on the contralateral side can lead to cortical hyperexcitability, triggering cortical spreading depression (CSD). CSD, in turn, activates central trigeminovascular neurons located in the spinal trigeminal nucleus, resulting in headache attacks. In summary, the mechanism of headache in Wallenberg syndrome remains unclear and largely speculative. In this case, the presence of right facial sensory disturbance suggested the involvement of the spinal trigeminal nucleus, implying that damage to this region may have induced hyperactivation of the trigemino-autonomic reflex, which in turn activated the trigeminovascular system, triggering the headache.

The absence of a high signal on diffusion-weighted imaging (DWI) on the day of onset and the following day highlights the challenges in the early diagnosis of Wallenberg syndrome. A study by Seo et al. reported that approximately 50% of acute lateral medullary infarctions within 24 hours of onset were false-negative on initial DWI [5]. Possible explanations include that the cerebral blood flow may have fallen below the threshold for symptom manifestation yet remained above the threshold for diffusion restriction [14]. This means that small infarcts or the timing of imaging can make early detection on DWI challenging [15]. In this case, the infarction was detected on DWI on the sixth day after onset, emphasizing the importance of repeated MRI examinations when clinical symptoms persist, even if the initial MRI is negative. Although there have been reports of Wallenberg syndrome presenting with headache, cases in which thunderclap headache is the sole initial symptom, without arterial dissection and with a negative initial MRI, are relatively rare. This case represents a valuable example of an atypical clinical course of Wallenberg syndrome.

## Conclusions

We report a rare case of Wallenberg syndrome presenting with thunderclap headache as the main symptom, without findings of arterial dissection, and with two initial MRI scans failing to detect the lesion. This case suggests that thunderclap headache can be an initial symptom of Wallenberg syndrome and should be considered a differential diagnosis. Furthermore, headaches in Wallenberg syndrome may occur through non-dissecting mechanisms. Further evaluation is needed to elucidate the mechanisms underlying headache as a possible initial symptom. Even if the initial MRI is negative, the full spectrum of symptoms may take time to manifest, requiring careful diagnostic consideration. In particular, for patients with neurological abnormalities that are gradually becoming apparent, serial clinical and neuroradiological monitoring is essential.
